# The resilience of integrated community case management in acute emergency: a case study from Unity State, South Sudan

**DOI:** 10.7189/jogh.08.020602

**Published:** 2018-12

**Authors:** Naoko Kozuki, Katja Ericson, Bethany Marron, Yolanda Barbera Lainez, Nathan P Miller

**Affiliations:** 1 Research, Evaluation, and Learning Unit, International Rescue Committee, Washington, D.C., USA; 2 Health Unit, International Rescue Committee, New York, New York, USA; 3 UNICEF, New York, New York, USA

## Abstract

**Background:**

An active conflict in South Sudan in late 2013/early 2014 displaced approximately 2 million people over the course of several months. In May 2015, the International Rescue Committee and UNICEF conducted a mixed-methods case study of the impact of that acute emergency on integrated community case management (iCCM) of childhood illness programming in Payinjiar County, Unity State. The objective was to document the operations of an iCCM program during an acute crisis and to assess the program’s ability to continue operations.

**Results:**

This mixed-methods case study is comprised of semi-structured interviews and focus groups with key stakeholders such as policymakers, program implementers, community health workers (CHWs), and caregivers on their experience with iCCM programming during this time period. Routine program data were also analyzed to assess the effect of the crisis on key health indicators.

**Findings:**

Internally displaced persons (IDPs) nearly doubled the population in Payinjiar. Some displaced CHWs continued to provide treatment in host communities when they were able to take supplies with them. Despite no formal community mobilization effort by the iCCM program, many IDPs identified CHWs in the communities they were displaced to and obtained care from them. Caregivers who had been internally displaced reported preferring care from CHWs especially in contrast to risking an insecure journey to health facilities. The total number of treatments provided per month by CHWs dropped during the acute crisis, but recovered to pre-crisis levels within six months. CHW supervisors attempted to continue supervision by utilizing their networks to track down displaced CHWs and assess the security situation prior to visits. The monthly supervision rate dropped to the lowest level of 77% in February 2014, but rebounded to 91% by August 2014. Several CHWs and community leaders qualitatively validated this claim of sustained supervision.

**Conclusions:**

CHWs, including those who were internally displaced, continued to provide treatment for childhood illnesses during an acute emergency, and service provision recovered faster to pre-crisis levels than the formal health sector. International donors and humanitarian actors should recognize iCCM as a potentially high-impact humanitarian response. Flexible funding from donors would enable further evidence generation on iCCM approaches and improvements that could both sustain and enhance programming in acute crisis.

Despite global reductions in under-five mortality, 5.6 million children still die each year predominantly from preventable causes [1]. Fragile states only accounting for 12% of under-five children in low- and middle-income countries (LMICs), but they account for 22% of the under-five deaths. Furthermore, a majority of the ten countries with the highest under-five mortality rates is considered fragile [1]. A key reason for persistently high mortality rates is a population-wide lack of access to basic and essential primary care [2]. Integrated community case management (iCCM) of childhood illnesses, a strategy that deploys community health workers (CHWs) to provide treatment for uncomplicated cases of childhood pneumonia, diarrhea, and malaria, has been promoted as a way to increase access to life-saving treatment [3].

CHWs may play a unique role in emergencies by providing critical health care in the context of increasing morbidity and mortality and reduced access to care. Strong community health platforms may strengthen resilience, or the ability to recover and speed to recovery after onset of manmade or natural emergency [4]. There are a small number of documented experiences of community health services being delivered during emergencies [5-7], but limited detail is provided on how services were delivered, how the emergencies impacted service delivery, or on the effectiveness of the services.

In 2005, the International Rescue Committee (IRC) became the first non-governmental organization (NGO) to introduce iCCM to South Sudan, then southern Sudan, with Payinjiar County, Unity State as one of the first pilot implementation areas. Unity State is approximately 38 837km^2^ in size, had a 2015 population estimate of 74 000, and is mainly comprised of two ethics groups of Nuer and Dinka [8]. About 30% of that population is encompassed in the geographic areas discussed in this study. iCCM in Payinjiar has been implemented in parallel to and complementary of the IRC’s longstanding primary health care programming which included support to the local MoH primary health care centers (PHCCs) and units (PHCUs) since 1995. Payinjiar is rural and predominantly occupied by agricultural and pastoral Nuer tribal communities at the southernmost tip of the state. Payinjiar experiences prolonged periods of annual flooding that isolates communities, constrains access by vehicles, and causes regular displacement and food insecurity. Payinjiar borders the predominantly Dinka-inhabited Lakes State to the west and south, predominantly Nuer-inhabited Leer County to the north, and two counties of the ethnically divided Jonglei state to the East (see Figure S1 in **Online Supplementary Document(Online Supplementary Document)** for map). The geographic description here is based on administrative delineation at the time of the study, not the time of publication.

iCCM treatment services in Payinjiar are delivered by Community Based Distributors (CBDs, South Sudan’s CHW cadre providing iCCM services). The majority of CBDs are illiterate and female, considered volunteers, are nominated by the communities in which they reside, receive non-monetary (in-kind) incentives for their participation, and serve between 20 to 40 households within their home community. Each CBD maintains a box of supplies which includes a month’s supply of drugs, assessment and diagnostic tools (timer, counting beads, etc), visual aids, patient register, handbook with pictorial representations of their treatment protocols, and basic supplies such as scissors and pens.

CBD supervisors are paid IRC staff members recruited from the catchment areas they are assigned to supervise, oversee between 15 to 20 CBDs, and move throughout the county primarily on foot. Supervisors act as the link among CBDs, the communities, and the IRC. Supervisors’ catchment areas are organized by the local MoH PHCCs and PHCUs. They are responsible for the monthly distribution of drugs and supplies to CBDs from small stocks maintained at the local facility and/or their homes and mobilization of CBDs for monthly meetings or trainings. They also conduct one supervision visit per month to monitor drug levels, experienced stockouts and the quality of the CBD’s work with a brief assessment. Due to the distance between villages, Supervisors on average travel on foot up to four hours to visit one to two CBDs daily. On a monthly basis, supervisors support CBDs to complete their patient registers which are collected and compiled into an aggregated report, complete supervision checklists documenting the results of their visits, and travel up to eight hours on foot to reach the IRC’s office in Ganyliel to attend monthly meetings to submit reports, collect needed supplies and drugs for their teams, and coordinate upcoming activities with the program management team.

At the time of the conflict, the IRC was also supporting the Payinjiarcounty health department to operate nine PHCUs offering limited services and one PHCC providing more comprehensive care. However, due to persistent challenges such as staffing shortages, drug stockouts, and distance from catchment communities, the iCCM program regularly provided more treatments for childhood illnesses in Payinjiar than these facilities.

The IRC and UNICEF conducted a case study on the IRC’s iCCM programming in Payinjiar County, Unity State, focusing on the perior of late 2013/early 2014, a time of acute crisis. Payinjiar County had a population of approximately 60 000 in 2014 [9]. In December 2013, heavy military exchanges between government (primarily Dinka) and opposition forces (primarily Nuer) erupted in the capital, Juba, ignited by a power struggle between President Salva Kiir and his former deputy Riek Machar. Latent historical tensions along tribal lines ignited throughout the nation with clashes of opposing armed forces moving out of Juba and into rural areas of Unity, Jonglei, and Upper Nile states. Over 1.35 million people were internally displaced and an additional 460 000 people fled to neighboring countries [10]. The conflict reached northeast areas of Payinjiar County in February 2014 as government forces pushed into the opposition county from bordering Dinka regions. Clashes between the government and opposition forces moved quickly through Unity State; most active violence subsided within one month. However, an estimated 35 000-40 000 internally displaced persons (IDP) had fled or arrived in Payinjiar from inside the county as well as from neighboring counties and states [11]. The IDPs, who were predominantly Nuer, established informal settlements in areas deemed safe due to the protective swamps and rivers in addition to integrating into host community villages where possible. By July of 2014, approximately 260 000 IDPs sought refuge throughout Unity State (see **Figure 1**) [12]. More details on the conflict are available elsewhere [10,13].

**Figure 1:**
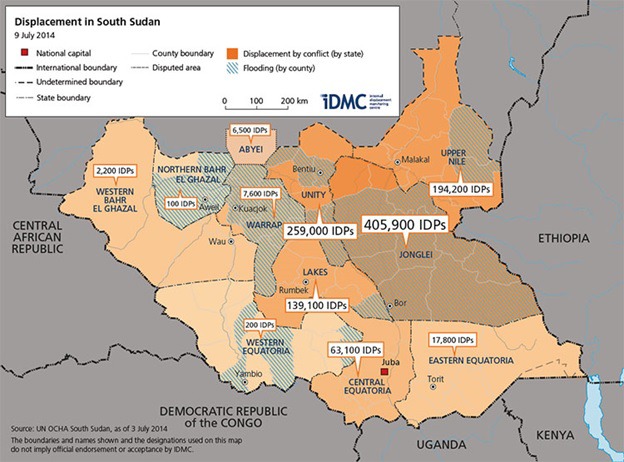
Displacement in South Sudan, 2014 [12].

During the acute crisis period, the iCCM program continued its operation following routine protocols, with minor adjustments to accommodate for access and mobility constraints related to insecurities. Some activities requiring mobilization of CBDs like meetings and trainings were suspended. Supervisors in high risk areas were encouraged to utilize guidance from local leaders to inform the safety and necessity of their movement to CBD homes or across greater distances to the IRC office; reporting periods were extended to allow flexibility for mobility-related delays in Supervisors’ collection and submission of monthly reports should they be unable to reach the IRC office by regular deadlines.

The objectives of the study were to document the effect of acute crisis on iCCM program implementation, and to extract lessons learned for future community health programming in fragile and conflict-affected settings.

## METHODS

The case study consisted of qualitative data collection conducted in May 2015 and quantitative analysis of routine program data from December 2012 to January 2015.

### Qualitative data collection

Semi-structured in-depth interviews (IDIs) and focus group discussions (FGDs) were conducted with key iCCM stakeholders. Four payams (payam being an administrative unit) out of ten in Payinjiar County were purposively selected as sites for data collection for maximum variation, based on magnitude of in- and out-migration, active conflict, and destruction caused by the conflict. The estimated population of 21 000 people at the time of the study [8]. Details on payam selection are available in Appendix S2 of **Online Supplementary Document(Online Supplementary Document).**

#### IDIs

All interviews were conducted in May 2015 with three general categories of informants: 1) policymakers (national, state, and county), 2) program implementers (national, state, or county), and 3) health workers in PHCCs and PHCUs primary health care centers (referral facilities) (**Table 1**). Respondents were purposively selected based on their ability to provide varied perspectives on research questions. Separate interview guides were developed for each type of informant.

**Table 1:** Description of qualitative data collection

Group	Research topics	Methods	Intended sample size	Actual sample size
Policymakers	1. The policy environment for iCCM	IDI	8-10	6 NGO implementing partners, 2 national-level MoH, 1 Unity State MoH official, 1 CDH County MoH staff
	2. The health system structure and functionality			
	3. Integration of iCCM into the national health system			
	4. Sustainability of iCCM			
	5. Challenges to implementing iCCM in the country			
	6. How the emergency affected the health system			
	7. How the emergency affected iCCM support			
	8. What was done to improve health services in response to the emergency			
	9. How the program could be adapted to better prepare for or respond to the current emergency or future emergencies			
Program implementers	1. Details of the iCCM program and the challenges they faced	IDI	5-8	5 IRC field-based program staff
	2. How they responded to challenges			
	3. The impact of the emergency on the population in affected areas			
	4. The impact of the emergency on the iCCM program and what was done to respond to these challenges			
	5. How the program could be adapted to better prepare for or respond to the current emergency or future emergencies			
Health workers	1. How the health facility supports iCCM	IDI	2-3	2 health workers at local PHCUs
	2. Details of the challenges they faced in supporting iCCM			
	3. The impact of the emergency on health facility services and support to the iCCM program and what was done to respond to these challenges			
	4. How to improve health services and support to iCCM in emergencies			
CBD supervisors	1. Details of their work and the challenges they faced	FGD	2	3 IDIs, 2 FGDs
	2. The impact of the emergency on their work and what was done to respond to these challenges			
	3. How the program could be adapted to better prepare for or respond to the current emergency or future emergencies			
CBDs	1. Details of their work and the challenges they faced	FGD	2	3 FGDs
	2. The impact of the emergency on the community			
	3. The impact of the emergency on their work and what was done to respond to these challenges			
	4. How to improve their ability to provide services during the current or future emergencies			
Community leaders	1. Impressions of iCCM services	FGD	2	4 FGDs
	2. The impact of the emergency on the community			
	3. The impact of the emergency on availability of iCCM services			
	4. What was done to improve availability and provision of iCCM services during the emergency			
	5. How to improve availability and provision of iCCM services during the current or future emergencies			
Caregivers	1. Impressions of iCCM services	FGD	3	4 FGDs
	2. The impact of the emergency on the community			
	3. The impact of the emergency on availability of iCCM services			
	4. What was done to improve availability and provision of iCCM services during the emergency			
	5. How to improve availability and provision of iCCM services during the current or future emergencies			

iCCM – I ntegrated Community Case Management, CBD – community-based distributor, CDH – County Health Department, FGD – focus group discussion, IDI – indepth interview, IRC – International Rescue Committee, NGO – non-governmental organization. MoH – Ministry of Health, PHCU – primary health care unit

#### FGDs

All FGDs were conducted in Payinjiar in May 2015 with the following groups: caregivers, CBDs, CBD supervisors, and community leaders. Ten CBDs from each of the targeted payams were randomly selected. Caregivers were mobilized by iCCM CHD officers, county Ministry of Health (MoH) officials, and CBD supervisors in each selected area on the day of the FGD. The caregiver FGDs were comprised of female participants; eligible participants had to have utilized CBD services in the recent past and have children under five years of age to ensure caretakers would be positioned to address research questions focused on how the emergency affected available CBD services, given their previous knowledge and experiences. Community leader participants were recruited by the iCCM Officer and CBD supervisors from a central area in the selected payams where leaders congregate. The categories of respondents and topics for both IDIs and FGDs are summarized in **Table 1**.

#### Data collection

Guides were composed in English and translated into Nuer, then back-translated to check accuracy. The guides were subjected to several rounds of pre-testing and revision. Two Nuer-native translators from Payinjiar County and three expatriate researchers from Forcier Consulting, a private research firm, served as qualitative data collectors.

The IDIs and FGDs were conducted in Payinjiar by two teams, each consisting of a researcher from Forcier Consulting and a Nuer interpreter. Two IRC staff provided technical oversight of the FGDs for quality assurance and only attended FGDs that did not feature IRC-funded staff. The third Forcier Consulting researcher conducted all IDIs with national-level informants in Juba in English. IRC representatives did not attend any IDIs.

Due to staff concerns regarding the sensitivity of recording conversations in a conflict context, all the Nuer-language interviews were translated verbally in real-time. Notes were taken by hand by the Forcier researcher and later typed. For English-language interviews, notes were taken in English by the researcher. Each day, the team debriefed on any challenges during data collection.

Informed consent was obtained from all respondents. Ethical approval was received from the Institutional Review Board of South Sudan MoH.

#### Data analysis

Data analysis was conducted using a combination of deductive and inductive methods. Initial codes were developed to represent higher-level themes based on the research questions: 1) background, 2) challenges of implementing iCCM prior to the crisis, 3) impact of the conflict, 4) response to the challenges created by the conflict, and 5) recommendations regarding response to conflict or general health service provision. Within each of higher-level theme, sub-themes were identified inductively from the data. The data were coded by hand, once by an IRC focal point and once by a UNICEF focal point independently, and organized into an Excel matrix (row for theme, column for each interview or FGD). The primary author used both sources to summarize the major themes.

### Quantitative data collection

IRC’s routine iCCM program data, covering activity from December 2012 to January 2015, were examined to assess the effect of the crisis on key indicators.

#### Data collection

The routine monitoring data collection system was as follows. CBDs entered data into a Patient Register that captures data on name, sex, age (2 to <11 months vs 12 to <59 months), assessment and classification of illness, treatment given, and whether the child was referred to a health facility. CBD Supervisors used monthly reporting tools that aggregated the aforementioned information, along with drug stock availability at the CBD level. The aggregated data were reviewed and verified by iCCM Program and Monitoring and Evaluation Officers and entered into the program’s District Health Information System (DHIS) database. No changes to the data collection system or tools were implemented in response to the crisis or following the emergency phase. Supervisors and program staff continued to follow the standard protocols and procedures for monthly and quarterly reporting periods. The data elements extracted for this study are listed in Supplemental Table 1. Population estimates for host and IDP communities were taken from the Initial Rapid Needs Assessment mechanism (example report is cited here [14]) and data from World Food Program and the South Sudan National Bureau of Statistics [15].

Other indicators (see **Table 2**) were calculated using the data elements from Supplemental Table 1 to describe utilization, access, and coverage. All data points were exported into Microsoft Excel and pivot tables were created for each indicator and summarized by month. Some indicators were compared against relevant DHIS data from health facilities in Payinjiar County that were supported by the IRC at the time. Monthly data on IRC’s Payinjiar County warehouse stock of artemisinin-based combination therapy, oral rehydration salts (ORS) and amoxicillin and CBD stockout rates were also available. Based on previous programmatic experience however, the quality of the stockout data is questionable.

**Table 2:** Indicators constructed using available data elements

Indicator	Numerator	Denominator
CBD reporting rate	Number of CBDs who submitted a monthly report	Number of active CBDs in the catchment area
Estimated number of child contacts with a CBD, per child per year*	Number of under-five children seen by CBDs, times 12 (to derive annual rate)	Estimated total under-five children in catchment area
Treatment rate (treatments per child per year)*	Number of treatments given for presumptive malaria, diarrhea, and/or pneumonia, times 12 (to derive annual rate)	Estimated total under-five children in catchment area
Supervision rate	Number of CBDs who received a supervision visit	Number of active CBDs in the catchment area
Under-five contacts per CBD per month	Number of under-five children seen by CBDs	Number of CBDs who submitted their monthly report
Referral rate	Number of under-five children referred to a health facility	Number of under-five children seen by CBDs in catchment area

CBD – community-based distributor

*These indicators are reported based on monthly data, but presented as an annual rate, to match routine indicators used in iCCM reporting.

## RESULTS

For Payinjiar County during this period, a monthly average of 262 CBDs were documented as active (trained, working, and reporting at least once in a four-month period). A total monthly average of 239 CBDs reported data between December 2012 and January 2015, with an average monthly reporting rate of 92%. For the four payams selected for this study (Ganyiel, Pachak, Payinjiar, and Thornom) a total average of 123 CBDs submitted monthly reports out of a total 136 active CBDs (91%). During the crisis period, CBD supervisors from the selected payams followed the routine reporting procedures to collect monthly reports from the majority of their CBDs, with an average of approximately 13 CBDs each month unable to report. The furthest right column of **Table 1** describes the qualitative data collected.

### Effect of the crisis on iCCM programming and subsequent response

#### Population displacement

Payinjiar County experienced a significant influx of IDPs beginning in December 2013 and primarily lasting through the first quarter of 2014. The influx nearly doubled the estimated county population from 44 224 to a total of 83 433 [16]. Some Payinjiar County residents were displaced as well.

Caregivers, CBDs, and CBD supervisors shared painful accounts from the acute emergency. One caregiver recounted, “*I moved with the healthy children when the fighting came. We left the lame and sick children behind. We all lost family, people starved to death, things were burned, we fled our homes. We left with nothing, now we have nothing.*” Another caregiver shared, “*People died from hunger, even if you managed to run with your child, many* [later] *died from hunger. There are also other dangers, for example from wild animals. Many people died.*”

Additionally, during 2014 and most of 2015, Payinjiar County was classified at critical levels of food insecurity at phase 3 (crisis) or phase 4 (emergency) [16]. CBDs were reported as having spent more time outside the home in search of food and water for their own households. An IRC program staff member reported, “*Issue of food security was drastically affected by conflict. People were not able to cultivate, had to travel farther to find food. CBDs are not at home.*” CBDs recalled an increase in the number of malnourished children. One caregiver noted, “*Children eat without milk, [which is] causing a lot of diseases...Another* [is] *we don’t have enough food. [We are] now eating grass.*” CBD supervisors faced challenges finding CBDs for regular supervision visits as they were more frequently absent.

#### Continued care through displacement

Displaced CBDs were occasionally tracked down by their CBD supervisors and home community members to notify them when it was safe for them to return. This often required supervisors from different catchment areas to travel to and communicate with each other if a CBD was displaced to another supervisor’s area of supervision. CBD supervisor reported, “*The community worried a lot about their children when the CBD ran away… No iCCM activities continued until they came back. Only CBDs know how to treat a child.*” A respondent within the national MoH recognized, “*The availability of the iCCM services was not affected so much by the conflict. CBDs tend to move with their communities when these move and they take their boxes with them. Wherever the community settles, the CBDs continue to provide services to their communities.*”

The reporting rate of CBDs to their supervisors dropped from 93% in December 2013 to the lowest value of 77% in February 2014, but a majority of the CBDs reported activity throughout the crisis period, and the rate only recovered from there (Figure S2 in **Online Supplementary Document(Online Supplementary Document)**).

#### CBD utilization

**Figure 2:**
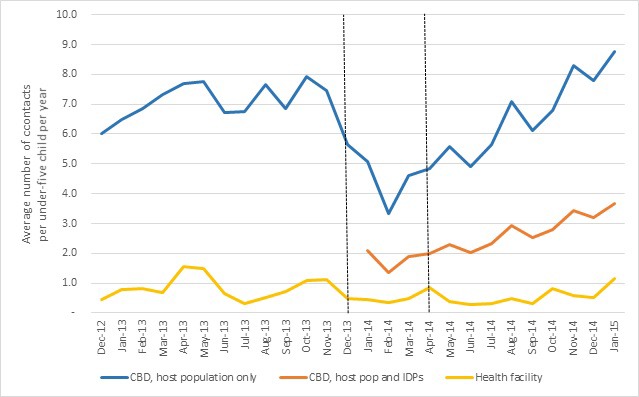
Average number of contacts per under-five child per year in four payams of Payinjiar County, Dec 2012-Dec 2014. The area between the vertical lines roughly represent the period of acute crisis.

Using the host population as the denominator, an under-five child had on average 5.7 contacts with a CBD per year in December 2013. This dropped to the lowest value of 3.3 contacts in February 2014, but recovered to 5.6 contacts by May 2014. When including the estimated influx of IDPs in the denominator, the rate was lowest in February 2014 at 1.4 contacts per child per year. The number of contacts with CBDs was consistently higher than with health facilities (**Figure 2**).

#### CBD treatment

**Figure 3:**
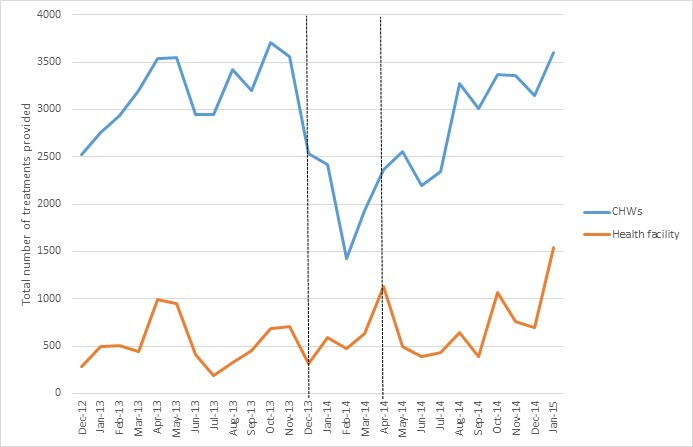
Total number of treatments provided to under-five children by CBDs in four study payams and in the nearest health facility (encompassing greater catchment area), Dec 2012-Dec 2014. The area between the vertical lines roughly represent the period of acute crisis.

The total number of treatments provided by CBDs is displayed in **Figure 3**. While a drop in the number of treatments was seen around February 2014, the total number recovered to pre-crisis levels within a few months. The number consistently remained higher than health facilities.

**Figure 4:**
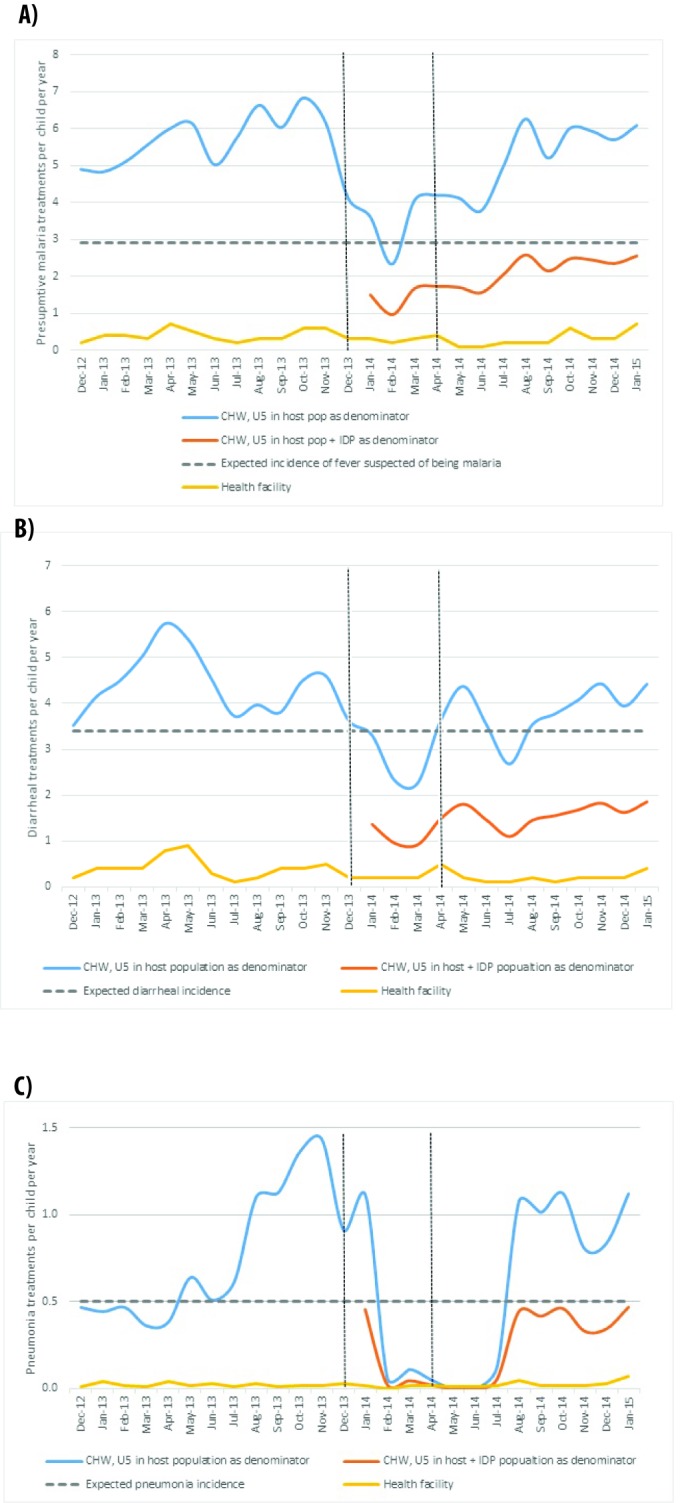
**A)** Presumptive malaria treatments per under-five child per year, provided by CBDs and health facilities. **B)** Diarrhea treatments per under-five child per year, provided by CBDs and health facilities. **C)** Pneumonia treatments per under-five child per year, provided by CBDs and health facilities. The area between the vertical lines roughly represent the period of acute crisis.

Treatment rates (number of treatments provided per under-five child per year) are displayed in **Figure 4** (panels A-C) by illness, plotted with a horizontal line representing the expected incidence per child. Given that South Sudan-specific estimates were not available, the Uganda national estimate of 2.9 cases per child per year was used as a proxy for fever suspected as malaria [17], and the Sudan estimates of 3.4 cases [18] and 0.5 cases [19] per child per year were used for diarrhea and pneumonia respectively.

Assuming a catchment area only consisting of the host population, CBDs’ treatment rate of fever suspected of being malaria dropped below the expected incidence of fever from December to March 2014, but otherwise recovered. When accounting for the influx of IDPs, CBDs presumed treatment rates per child per year were below the incidence of fever suspected of being malaria. (**Figure 4**, panel A).

A similar pattern was observed for diarrhea (treatment defined as receiving ORS, with or without zinc) (**Figure 4**, panel B). Assuming a catchment area only with the host population, CBDs’ treatment rate of diarrhea dropped below the expected incidence around February 2014 and again around July 2014 (perhaps due to ORS stockout, see Supplemental Table 2 in **Online Supplementary Document(Online Supplementary Document)**), but otherwise remained above the expected incidence. When accounting for the IDP influx, CBDs did not provide enough treatments to cover the expected incidence.

For pneumonia, almost no treatments were provided immediately after the crisis. The average number of treatments per child per year provided by CBDs before December 2013 was 0.74, above the expected incidence. From January-July 2014, this number dropped to 0.21 when accounting only for the host population or 0.09 when including IDPs. For August 2014-January 2015, the CBDs treatments recovered to 1.0 when accounting for the host population and 0.4 when including IDPs. The drop in pneumonia treatments between February and August also coincided with national-level stockout of amoxicillin. Treatment rates began to rise in August, after new stock was received (**Figure 4**, panel C).

For all three illnesses, treatment provided by CBDs remained higher than health facilities.

#### Preference for CBD care

Caregivers preferred seeking care from CBDs over risking an insecure journey to a potentially ill-equipped or unstaffed nearby PHCU for care; for many communities, the only PHCC was a half day’s travel by foot. Many caregivers believed that MoH facilities were non-functioning; one caregiver mentioned, “*the (referral facility) has been closed, the health workers all left, they did not want to be on the front lines.*” Based on available programmatic data, this was not entirely accurate. Three PHCUs located within the study payams had closed in July 2013, recently but before and unrelated to the crisis. The other facilities remained open even if at lower capacity with an additional mobile PHCU deployed in the study payam of Thornom (location of the largest IDP settlement) as part of IRC’s emergency response efforts in July 2014. A majority of caregivers believed that CBDs were their best option for care. Both community leaders and caregivers, as well as CBDs themselves, reported pressure on CBDs to provide treatment for the population beyond the age of five during the emergency phase. A CBD stated, “*only challenge is drugs* [are given only for children] *under five years downward. Big challenge is 16* [years] *and above,* [they] *come to us when sick,* [saying] *‘Why are you not giving us* [drugs]*?*’” Similarly, a caregiver criticized that “the CBD can help, but they only help children, not us older people.”

#### CBD workload

**Figure 5:**
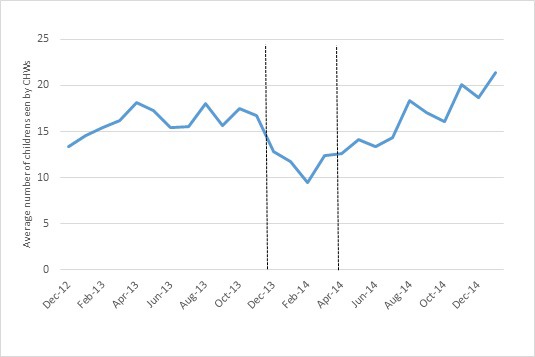
Average number of under-five seen per CHW per month. The area between the vertical lines roughly represent the period of acute crisis.

The average number of children seen by each reporting CBD dropped to approximately 10 children per month around the time of the onset of the conflict. The lowest caseload occurred in February 2014. The number increased from then, with roughly 21 children being seen per month by each active CBD in January 2015 (**Figure 5**).

#### Supply chain

Several interviewees reported the negative impact of the crisis on drug stock. One CBD supervisor reported that drugs used to be stored in large quantities in Ganyiel, but they no longer felt secure doing so with the threat of damage or looting. Many CBD supervisors maintained a practice of storing the stock for their catchment areas in the local health facilities or in their homes where they had easy access. Supervisors perceived other storage options as riskier, although the IRC’s drug warehouse in Payinjiar County was not looted during the study period. Drug stockouts at the warehouse level occurred occasionally due to the program’s inability to receive resupplies from Juba. According to warehouse data, the stock for ACT and ORS recovered within a few months, while zinc and amoxicillin stock remained low or out (Supplemental Table 2).

Numerous interviewees reported an increase in demand for CBD services with the influx of IDPs, which subsequently led to increased pressure on supplies. Several CBD supervisors indicated that stockouts were more frequent among CBDs working in areas with IDPs. Several CBDs, CBD supervisors, and caregivers indicated that stockouts were not a major concern prior to the start of the crisis. With increased frequency of CBD stockouts, some CBDs referred children to other CBDs who had drugs left.

#### Supervision

CBD supervisors faced several difficulties in continuing supervision, due to concerns of physical security and CBD displacement. CBD supervisors attempted to continue supervision by utilizing their networks to track down displaced CBDs and to assess the security situation prior to making supervisory visits. Several CBDs and community leaders validated this claim.

#### Health facility services

Health facilities were severely affected, with looting and damage to equipment and supplies. A Unity State MoH staff reported that “*…many health centers lost all equipment and facilities and some were burnt down. Human resources were strained, many health centers lost staff as people fled the area*.” Even facilities that remained opened or eventually reopened as part of emergency response experienced major stockouts. The pre-positioned drugs for the rainy season were also largely looted or destroyed.

Caregivers appeared less likely to complete referrals because of their concern that the referral facility would not be operating fully. A caregiver mentioned that some health facilities remained without drugs even after reopening and that “*we find out by walking there that there are no drugs. That is the only way to be sure.*” There were concerns expressed by caregivers of leaving other children behind in an insecure situation while taking the sick child to the facility.

#### Funding

Two major donors funded the consortium that IRC operated its iCCM program under, one of which also funded emergency relief efforts following the start of the conflict. IRC staff reported that at the onset of the crisis, one donor communicated hesitancy to continue funding large scale development-focused programming, such as iCCM, since resources could potentially be reprioritized to support emergency response interventions. Uncertainty of the funding stream caused the IRC to limit iCCM programmatic spending and rely heavily on unrestricted private funding to cover implementation costs. The security of iCCM funds was reestablished 2-3 months later. The potential for redistribution or cancelation of funds never came to fruition.

Staff from another implementing agency indicated that one donor “*was a very flexible donor to context, allowing the program to continue and trusting the implementing partners’ judgement to get things done. There were no other donors [to the iCCM consortium] which helped in allowing this flexibility.*” Another implementing agency indicated that “*funding did not significantly change despite the increased program needs and overburdening in some cases. It didn’t stop, which allowed the program to continue, but it also did not increase given the increased demand.*” Multiple donor representatives were contacted for interviews, but none were available.

### Recommendations from respondents

Many recommendations were not emergency-specific; there were requests for increased provision of drug supply (both at the county and CBD level), for a larger CBD cadre, and for the provision of or increase in incentives for CBDs and CBD supervisors including the distribution of equipment such as bicycles, flashlights, and satellite phones (for CBD supervisors). Caregivers expressed their desire to see the CBD treatment menu expanded to serve more ages and illnesses, and CBDs expressed willingness to expand the scope.

Both government representatives and implementing partners suggested strengthening community involvement in iCCM programs, as they perceived community ownership to be weak and strengthening this link can allow for maintenance of services despite disruptions. Another MoH staff member highlighted other local structures such as local chiefs, village elders, and church leaders needing to be engaged in the day-to-day running of the program. Many of these recommendations applied both to general iCCM programming, and its resilience in emergencies.

For future emergencies, several implementing partners suggested expanding the iCCM package to include additional components, such as rapid diagnostic tests for malaria, vitamin A supplementation, and deworming. An implementing partner as well as several CBD supervisors also suggested deploying mobile medical units with staff trained in iCCM treatment protocols in place of integrated management of childhood illness (IMCI), normally requiring medical staff with more advanced training to deliver. Implementing partners and CBD supervisors also suggested bringing storage facilities for drugs and supplies to the payam level for better pre-positioning.

An implementing partner emphasized the need for donor flexibility; donors need to recognize the balance of financial risk and provision of services in fragile contexts and be willing to pre-position supplies for emergencies, with the understanding that some may be lost.

## DISCUSSION

The retrospective case study observed that despite notable effects of crisis onset on service delivery and use, the iCCM program continued to operate and rebounded to pre-crisis levels within a few months. The delivery of iCCM services continued without formal mobilization of CBDs or their supervisors by the program implementer. While we cannot make definitive statements on the impact that the iCCM program may have had on the conflict-affected populations, our data hint that this program provided services to the most vulnerable populations during a physically and emotionally traumatic period. The effect may have been higher if the program had received emergency funding and also if CBDs had been formally prepared for acute emergency response. Given the challenges of conducting research in war-affected areas, this study is a unique addition to the knowledge gap in fragile- and conflict-affected contexts. The ability of this case study to also triangulate data strengthened our conclusions, allowing us to mitigate the effects of potential data quality issues attributable to data collection during time of crisis.

Community health programming is traditionally not considered an intervention for emergency response, but this case study provided evidence in the need to fund and support, rather than halt, community health programs during acute emergency. The results of this study are consistent with related studies of CHWs during the Ebola outbreak in Guinea, Liberia and Sierra Leone [20-22] and during floods in Bangladesh [23]. Those studies also showed that CHW services were more resilient during the emergencies than were health facility services, and also highlighted the need for continued support to CHWs, especially with regard to continued drug supply. Implementing agencies have failed to advocate effectively for funding iCCM in an emergency, partially because little evidence exists to support the implementation of iCCM in an active conflict or emergency setting. This case study highlights the missed opportunities in the intersection between the humanitarian and development fields.

Prior to the start of the acute crisis, CBDs supported by the IRC consistently provided treatment coverage that met the expected incidence of presumed malaria, diarrhea, and pneumonia, and exceeded the treatments provided at health facilities. During and immediately following the acute crisis, the iCCM program in Payinjiar County was able to continue providing services. Including the IDP inflow, the estimated number of CBD treatments per child per year during the acute crisis was well above mean treatment rates published for sub-Saharan Africa. In a study that synthesized data from 23 iCCM program evaluation and research projects, the mean treatment rate (cases treated per child per year) was 0.6 for malaria, 0.4 for pneumonia, 0.4 for diarrhea, and 1.4 combined [24]. Despite the emergency context, the CBDs in our study surpassed these values. Qualitative data noted that many CBDs continued to provide treatment, either in their home communities or in host communities if they were able to bring their supplies.

The sustained provision of care and resilience of the CBD system during a time of crisis highlighted the value of investing in community-based health systems. Furthermore, the structure of iCCM, which relies on being imbedded within the community, appeared to lend itself to quick adaptation to a population’s needs. The communities’ trust of CBDs as well as their name recognition drew demand for health services at a time when facilities were inaccessible or not operating. CBD services were even in high demand by IDPs who did not know the local CBD, but were informed of the CBDs’ availability.

Quantitative and qualitative data revealed drug availability as a significant limiting factor in the CBDs’ ability to service their communities. Analysis of warehouse stockout data confirms that the amount of drugs distributed did not change despite increased demand. Such data highlight the current program’s inability to respond to acute changes in service demands. Adequate supplies, including buffer stocks, faster feedback mechanisms for preventing stockouts and responding when stockouts occur, and flexible supervision may best prepare iCCM programs for times of crisis.

There are several limitations to our study. While we expect the quality of routine program data to be sufficient to capture trends, routine data from the crisis period likely have quality issues. CBD recall of demographics, treatments, and referrals may have been poor if not recorded immediately after service; the time lag of receiving assistance from a family member or CBD supervisor to document the information could have been longer than usual during the conflict. Stockout data are captured by supervisory staff and we note inherent flaws in this design; we expect inaccuracies due to supervisors’ perceptions and fears of stockouts directly linked to their own job performance and of misinterpretation of stockout as their inability to restock a CBD vs CBD-level stockout. Routine data collection procedures lacked feedback mechanisms to quickly capture and report true demand, including changes in utilization and service delivery. There were no recent official population data available. The 2008 census was the last census of reliable quality implemented by the government. For qualitative data, cultural sensitivities and available resources did not allow for full interview transcripts. The data collection occurred approximately 1.5 years following emergency onset, which may have introduced recall bias. Relatedly, the protracted state of emergency that lasted between conflict onset and time of data collection made it difficult for respondents to differentiate the period of “acute emergency.” There may have also been trauma associated with the emergency that prevented some interviewees from sharing their experiences.

## CONCLUSIONS

The iCCM program in South Sudan demonstrated resilience during an acute crisis. International donors and humanitarian actors should recognize community-based health care as a basic component of high-impact emergency preparedness and humanitarian response in situations such as that in South Sudan and advocate for flexible funding from donors to sustain and enhance community-based health care programming in emergency contexts. The sustained performance and natural adaptability of the CBDs’ services at the community level in South Sudan demonstrated such potential.
