# Supplementary material for: The resilience of integrated community case management in acute emergency: a case study from Unity State, South Sudan
Source: J Glob Health. 2018 Aug 24;8(2):020602. doi: 10.7189/jogh.08.020602 (PMC6119813; doi:10.7189/jogh.08.020602)

Online Supplementary Document

Kozuki et al. The resilience of integrated community case management in acute emergency: a case study from Unity State, South Sudan

J Glob Health 2018;8:020602

Supplemental Text 1: Description of South Sudan iCCM

Per MoH guidelines, CBDs are nominated by the community in which they reside and, during the study period from 2013-2014, had received non-monetary incentives, such as rain ponchos and umbrellas, but now receive monetary incentives. Each CBD is responsible for 20-40 households. For children 2-59 months, CBDs inquire about fever, diarrhea, and cough or difficulty breathing and the duration of those conditions, and also check for child's inability to drink or breastfeed, convulsions, stiff neck, chest in-drawing, lethargy, and vomiting. Any child with a danger sign, or with fever, diarrhea, and cough/difficulty breathing beyond a pre-determined number of days, or with bloody diarrhea are immediately referred to a health facility. CBDs provide artesunate-amodiaquine for the treatment of presumptive malaria (fever); low osmolarity oral rehydration salts (ORS) and zinc for diarrhea, and amoxicillin for presumed pneumonia.

Supplemental Text 2: Payam selection

The payams for inclusion in the study were selected by the IRC and UNICEF, in consultation with the iCCM program staff in Payinjiar County and Forcier Consulting. In Payinjiar County, there is a total of ten payams, in eight of which IRC implemented iCCM programming. A scoring system was created to determine the degree to which each payam was affected by the crisis and relevant data were collected to categorize the payams, with a higher score representing less stability: out and in migration of IDPs (max 2 points, 1 per type of IDP movement), active fighting or conflict (1 point), and destruction caused by the conflict (1 point). To capture the different experiences across the county, four payams were purposively selected based on ratings of the four descriptions listed: one with high ratings for all categories (Pachak Payam – rating of 4), one with low ratings for all categories (Ganyliel Payam – rating of 1), two with medium ratings for different reasons – one with out-migrations, fighting and destruction (Payinjiar Payam – rating of 3) and one which experienced significant in-migration (Thornom Payam – rating of 2). See table below for scoring.

The two medium-rated payams were selected over other medium-rated payams based on additional criteria. Kol Payam also scored a 3, but was not selected due to prohibitive physical distance from the IRC field office and inaccessible road conditions. Payinjiar Payam was prioritized over Pachienjok Payam because it was the second furthest area after Kol and therefore offered another factor of interest, as well as the "destruction" reported related directly to CBDs supplies being looted and damaged in addition to the community-level destruction of property and homes. Tiap Payam scored a 2 along with Thornom; however Thornom was prioritized due to its higher volume of IDP influx and it having remained a consistent location where IDPs settled over the course of 2014. Lastly, both Ganyliel and Pachar Payam scored a 1; Ganyliel was

prioritized due to its score relating to IDP in-migration and this payam having received the largest number of IDPs across all payams at the start of 2014.

Description of Payam Rating and Selection Process for targeted study areas

Payam name	Total Score	Migration of IDPs		Active Fighting/ Conflict	Destruction
		Out	In		
**Pachak**	**4**	**1**	**1**	**1**	**1**
Kol	3	1	0	1	1
Pachienjok	3	1	0	1	1
**Payinjiar**	**3**	**1**	**0**	**1**	**1**
**Thornhom**	**2**	**1**	**1**	**0**	**0**
Tiap	2	1	1	0	0
**Ganyliel**	**1**	**0**	**1**	**0**	**0**
Pachar	1	0	0	1	0

*Selected payams in bold.

Supplemental Table 1: Quantitative data elements used for this study

	Data element	Data source
**Descriptive statistics**	- Year/month of iCCM implementation<br>- State/county/payam/catchment area	iCCM DHIS
	- Estimated total under-five children in catchment area*	2008 South Sudan Census Report [9]**
**Routine data**	- Number of active CBDs	iCCM DHIS
	- Number of CBDs who submitted a monthly report	iCCM DHIS
	- Number of CBDs who received a supervision visit	iCCM DHIS
	- Number of children seen by CBDs	iCCM DHIS
	- Number of children referred by CBDs to a health facility	iCCM DHIS
	- Treatment for under-five children by CBDs	
	o Number of ACT treatments given	iCCM DHIS
	o Number of ORS treatments given	iCCM DHIS
	o Number of zinc treatments given	iCCM DHIS
	o Number of amoxicillin treatments given	iCCM DHIS
	- Treatment for under-five children at health facility	
	o Number of treatments given for presumptive malaria	HF DHIS
	o Number of treatments given for diarrhea	HF DHIS
	o Number of treatments given for pneumonia	HF DHIS

*To estimate under five population targeted by the iCCM program, the IRC uses the estimated population projections (described in footnote 4) and applies a 20% estimate of the total estimated population per payam.

** The last and most reliable census conducted by the South Sudan National Bureau of Statistics was conducted in 2008. No other census was conducted that captured reliable population data; neither does any other population estimates provide population statistics disaggregated to payam level. It is common practice in South Sudan for implementing actors to utilize the 2008 population data as a basis with which to use an annual 3% population growth estimate across years to estimate population demographics in target catchment areas. This estimation is assumed to represent the host population in the county prior to any displacement or migration in 2014.

Supplemental Table 2: Monthly closing balance at warehouse of drugs

	ACT	amoxicillin	zinc	ORS
Apr-13	13320	1525	27750	11426
May-13	7486	1525	23564	0
Jun-13	5249	722.5	420	15840
Jul-13	N/A	N/A	N/A	N/A
Aug-13	N/A	N/A	N/A	N/A
Sep-13	9716	25080	0	18867
Oct-13	9338	18303	0	11763
Nov-13	616	14373	0	3763
Dec-13	616	9753	0	2763
Jan-14	7630	0	0	0
Feb-14	17005	0	0	0
Mar-14	18165	0	10000	0
Apr-14	15575	0	4900	6318
May-14	10200	0	900	0
Jun-14	6860	0	580	0
Jul-14	27335	1920	3950	24500
Aug-14	22455	210	420	9945
Sep-14	21856	66	2640	19000
Oct-14	17756	66	0	22417
Nov-14	12876	66	0	10922
Dec-14	15846	846	0	12820
Jan-15	11384	84	0	4417

Warehouse data were not available for months of July and August 2013

Supplemental Figure 1: Proportion of CBDs who submitted a monthly report in four payams of Payinjiar County, Dec 2012-Dec 2014

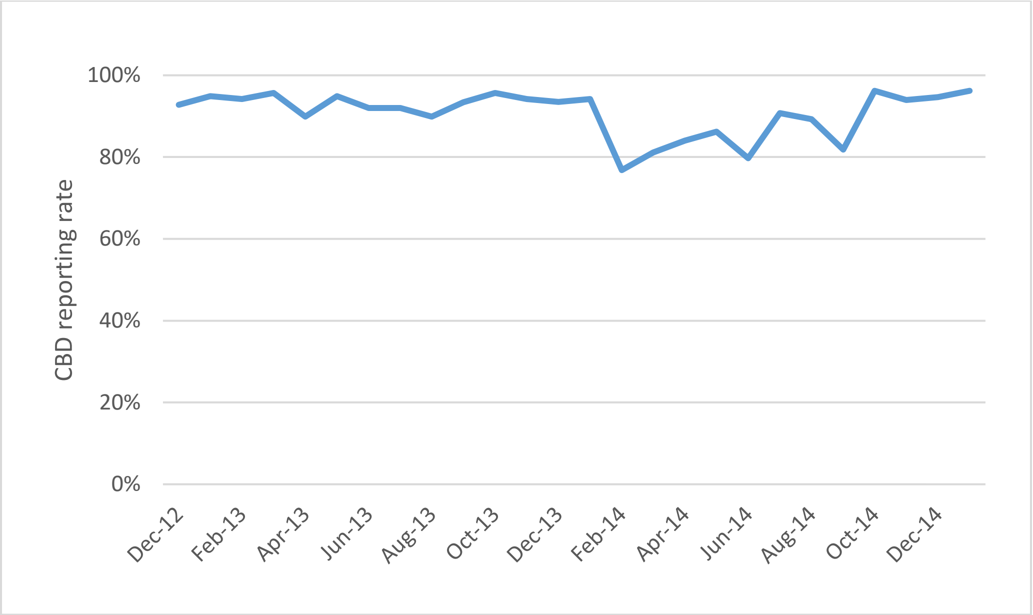

Supplement: Online Supplementary Document [file jogh-08-020602-s001.pdf]
